# The two-component signal transduction system and its regulation in *Candida albicans*

**DOI:** 10.1080/21505594.2021.1949883

**Published:** 2021-07-08

**Authors:** Biaoyou Liao, Xingchen Ye, Xi Chen, Yujie Zhou, Lei Cheng, Xuedong Zhou, Biao Ren

**Affiliations:** a State Key Laboratory of Oral Diseases & National Clinical Research Center for Oral Diseases& West China School of Stomatology, Sichuan University, Chengdu, China; b Department of Operative Dentistry and Endodontics, West China Hospital of Stomatology, Sichuan University, Chengdu, China

**Keywords:** *Candida albicans*, Phosphorelay, Histidine protein kinase, Response regulator, Two-component systems

## Abstract

*Candida albicans*, which can cause superficial and life-threatening systemic infections, is the most common opportunistic fungal pathogen in the human microbiome. The two-component system is one of the most important *C. albicans* signal transduction pathways, regulating the response to oxidative and osmotic stresses, adhesion, morphogenesis, cell wall synthesis, virulence, drug resistance, and the host–pathogen interactions. Notably, some components of this signaling pathway have not been found in the human genome, indicating that the two-component system of *C. albicans* can be a potential target for new antifungal agents. Here, we summarize the composition, signal transduction, and regulation of the two-component system of *C. albicans* to emphasize its essential roles in the pathogenesis of *C. albicans* and the new therapeutic target for antifungal drugs.

## Introduction

The infection and mortality rate of candidiasis have significantly increased in recent years due to tumor chemoradiotherapy, the widespread use of antibiotics, and the increase in the number of immunocompromised patients, such as those with HIV infection [1,2]. *Candida albicans* is the major pathogenic agent for candidiasis and one of the most common conditionally pathogenic polymorphic fungi from the human microbiome. It colonizes multiple ecological niches, including the oral cavity, reproductive mucosa, and the respiratory and gastrointestinal tract of healthy individuals. *C. albicans* can also cause cutaneous and mucosal infections such as thrush, vaginal infections, and life-threatening invasive infections [2,3]. Among the 20,788 isolates of invasive *Candida* collected from around the world for 20 years (1997–2016) in the SENTRY Antifungal Surveillance Program, 46.9% were *C. albicans* [4]. The incidence of *C. albicans*-induced candidaemia in China is 40.1% and up to 69.8% in Norway [5]. The proportion of *C. albicans* in ventilator-associated pulmonary candidiasis in ICU patients is as high as 46.36% [6]. *C. albicans* is even one of the most common coinfection fungi in COVID-19 patients [7].

The *C. albicans* colonization of different host niches depends on the capability to sense multiple environmental signals and then regulate its adaptation and switch between colonization and pathogenesis. *C. albicans* can transform reversibly between yeast, pseudohyphae, and hyphae forms, adapting to the stresses at different host niches and infected tissues under different conditions, including nutrition, pH value, temperature, oxidation, and immune status. *C. albicans* possesses a powerful signal transduction network, “the two-component system,” to continuously monitor the external environment and regulate its colonization and pathogenesis [8–11]. In the two-component system, the signal is introduced by the histidine protein kinase, and transferred through a series of phosphorylation events, finally phosphorylating the response regulator protein. Compared with the one-step transduction in the two-component system of prokaryotes, eukaryotes have a more complex multi-step phosphate transduction system. The two-component system in *C. albicans* regulates morphogenesis, responses to oxidative and osmotic stresses, quorum sensing, virulence regulation, etc. Here we summarize and discuss the structure and function of the two-component system in *C. albicans*, highlighting its role in pathogenesis and as a therapeutic target for new antifungal agents.

## The structure and signal transduction of the two-component system

**Figure 1.**
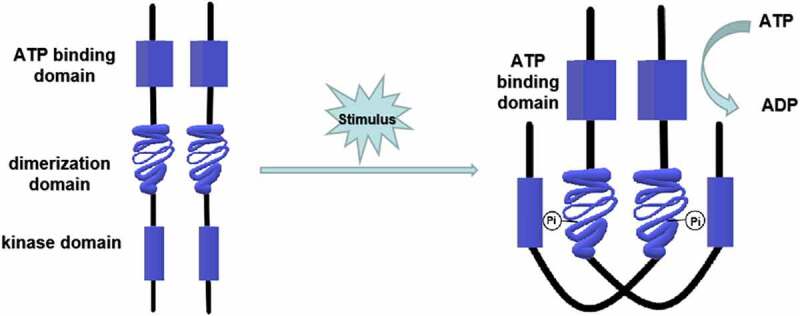
Structure and phosphorylation of HPK. The HPK is a dimer composed of two subunits. Each subunit contains an ATP binding domain, a dimerization domain, and a kinase domain (phosphorylation site). When the input domain of HPK is appropriately stimulated, the dimerization domain of one subunit will approach to the kinase domain of the other subunit to promote the phosphorylation

**Figure 2.**
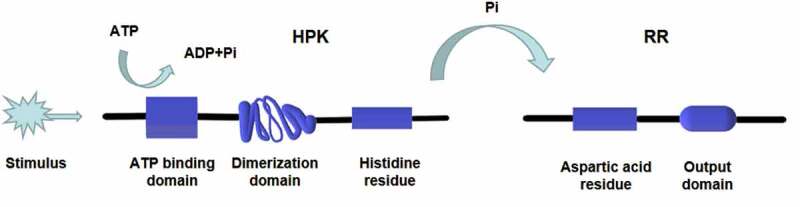
One-step phosphorylation of His-Asp in prokaryotes. A HPK is autophosphorylated on a histidine residue and the signal is subsequently transferred to a RR on an aspartate residue. The phosphorylated RR acts as a transcription factor regulating gene expression or a protein activity regulator. The transfer of phosphate acid from HPK to RR takes only one step (His-Asp)

Both prokaryotes and eukaryotes, including fungi, myxomycetes, and plants, contain the two-component signaling system [12–17], which can be divided into one-step and multi-step transduction modes. A typical two-component signaling system consists of a membrane-associated histidine protein kinase (HPK) and a response regulatory (RR) protein. The HPK is a dimer composed of two subunits, each containing an ATP binding domain, a dimerization domain, and a kinase domain (phosphorylation site). When the input domain of HPK is appropriately stimulated, the dimerization domain of one subunit approaches the kinase domain of the other subunit to promote phosphorylation [18] (Figure 1). The phosphorylation level of HPK affects the phosphorylation rate of the RR. Multiple HPKs might regulate one RR, or one HPK might regulate multiple RRs [19]. RR consists of a receiving module and an output domain. The receiving module regulates the output domain activity through the phosphorylation of aspartic acid residues (Asp). The output structure might be a transcription factor regulating gene expression or a protein activity regulator [18]. The two-component system was originally discovered by Ninfa and Magasanik et al. [20] in the nitrogen regulatory protein system of *Escherichia coli*. It is a typical one-step two-component system as an HPK is autophosphorylated on a histidine residue, and the signal is subsequently transferred to an RR on an aspartate residue (Figure 2). This nitrogen regulatory protein system of *E. coli* contains two proteins, NtrB (an HPK protein) and NtrC (an RR protein). NtrB catalyzes the transfer of a phosphate group to the aspartic acid of NtrC under nitrogen limitation conditions. The phosphorylated NtrC activates the transcription of nitrogen metabolism genes [12]. On the contrary, when the concentration of amine is too high, NtrB is regulated by upstream GlnD and PII proteins to promote the dephosphorylation and inactivation of NtrC, turn off the expression of genes encoding nitrogen metabolism-related enzymes, and stop the bacteria from absorbing nitrogen from the environment [21,22]. In the one-step two-component signal transduction system, the phosphate group is directly transferred from the HPK to the RR (His-Asp) [18].

**Figure 3.**
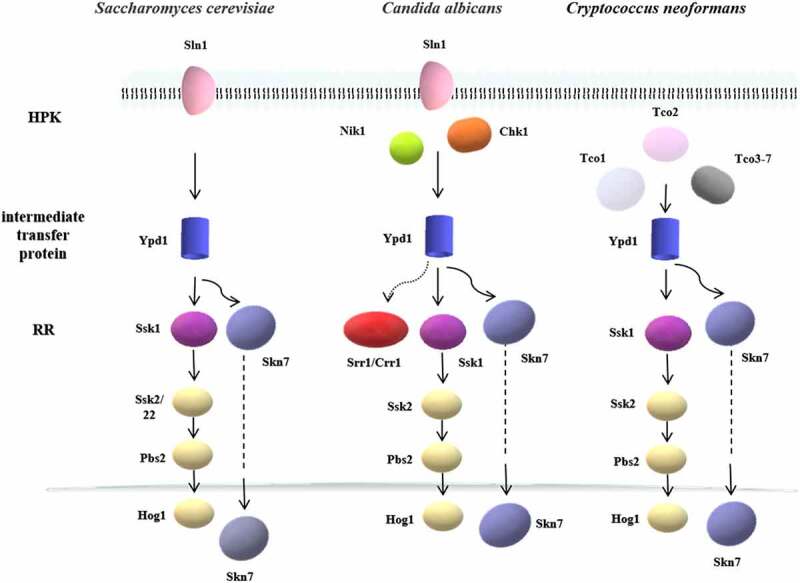
The two-component systems and the downstream pathways in different fungi. The two-component system in most eukaryotes is a multistep phosphate transduction model. The structure and conduction pathway of the two-component system are different in various fungi. For example, *S. cerevisiae* expresses only one HPK, *C. albicans* contains 3 HPKs, and *C. neoformans* has 7 HPKs. The phosphorylation level of HPK affects the phosphorylation rate of RR. Multiple HPKs may regulate one RR, while one HPK may also regulate multiple RRs

**Figure 4.**
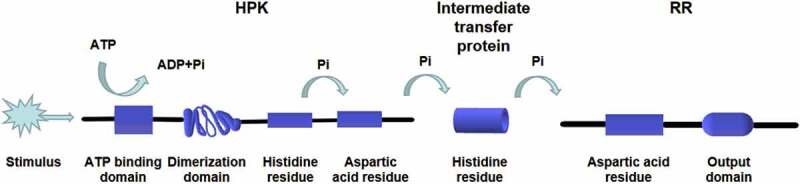
Multistep phosphorylation of His-Asp in fungi. After the HPK detects the stimulus signal, ATP is used as the donor to phosphorylate a conserved his residue. Subsequently, the phosphate group is transferred to the Asp residue of the same HPK receptor domain and then transferred to the Asp residue of the RR receptor domain through the his residue of intermediate transfer protein. Four phosphorylation events occur in sequence, forming the four-step phosphate transfer (His-Asp-His-Asp)

**Figure 5.**
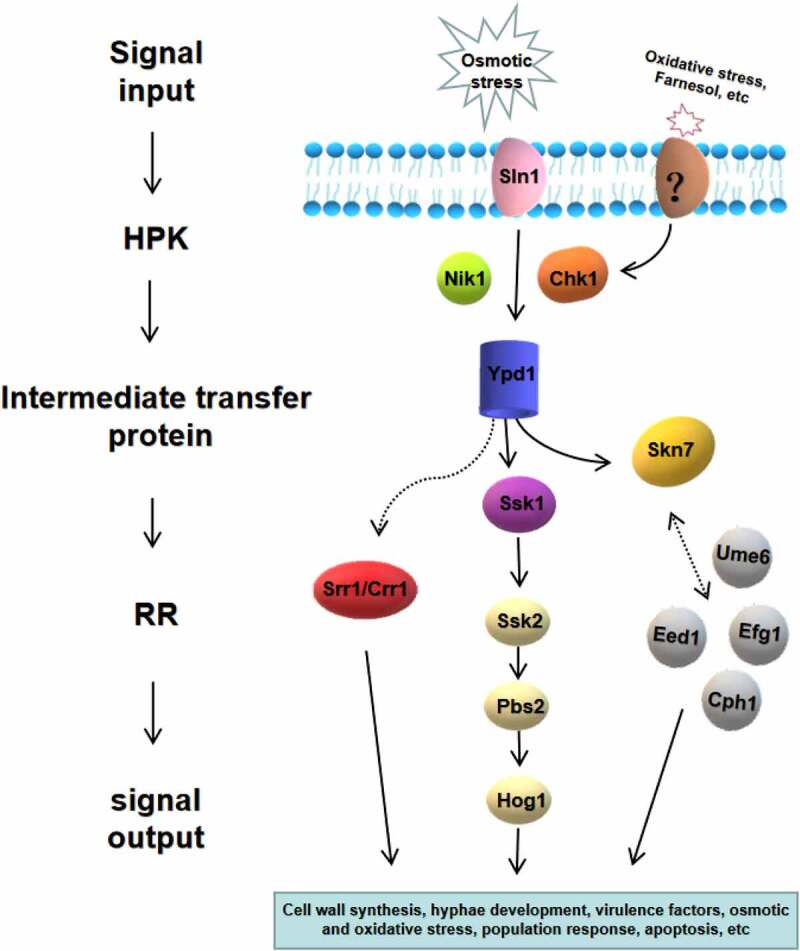
Two-component system of *C. albicans* and its downstream pathways. Seven proteins of the two-component system in *C. albicans* are shown, including three hybrid HPKs (Sln1p, Nik1p/Cos1p, Chk1p), three RRs (Ssk1p, Skn7p, Crr1p/Srr1p), and one intermediate transfer protein (Ypd1p). The downstream responses of two-component system are complex and diverse, which is highly related to morphogenesis, oxidative and osmotic stress, quorum sensing, virulence regulation and so on

In most eukaryotes, the two-component system is a multi-step phosphate transduction system [23–33] (Figure 3), usually consisting of a hybrid HPK, an intermediate transfer protein, and an RR. The structure and conduction pathway of the two-component system are different in various fungi. For example, *C. albicans* contains three HPKs [34], *Cryptococcus neoformans* has seven HPKs [32], and *Neurospora crassa* expresses eleven HPKs [35]. The transmission mechanism is as follows. ATP is used as the donor to phosphorylate a conserved His residue called H-box after the HPK detects the stimulus signal. Subsequently, the phosphate group is transferred to the Asp residue of the same HPK receptor domain, followed by being transferred to the Asp residue of the RR receptor domain through the His residue of intermediate transfer protein. Four phosphorylation events occur sequentially, forming the four-step phosphate transfer (His-Asp-His-Asp) system (Figure 4). The output components and processes of eukaryotic systems are more complex and diverse. For example, the two-component system and the downstream Hog1-MAPK pathway participate in signal transduction in *C. albicans* and other fungi [15,36], regulating the responses to oxidative and osmotic stresses, adhesion, cell wall synthesis, morphogenesis, and virulence [37–48] (Figure 5).

## Composition of the two-component system of *C. albicans*

Currently, seven proteins have been identified in the two-component system in *C. albicans* (Table 1), including three hybrid HPKs (Sln1p, Nik1p/Cos1p, and Chk1p), three RRs (Ssk1p, Skn7p, and Crr1p/Srr1p), and one intermediate transfer protein (Ypd1p) [49,50]. Ssk1p, Skn7p, and Crr1p/Srr1p are located within the cytoplasm, nucleus, and mitochondria, respectively [51–53].

**Table 1.** Components and functions of the two-component system of *C. albicans.*

**Composition**	**Species**	**Position**	**Feature**	**Function**				
				**Cell wall** **biosynthesis**	**Hyphae**	**Virulence**	**Osmotic stress**	**Oxidative stress**
**HPK**	Sln1p	Cytomembrane	Membrane protein	Yes	Yes	Yes	Yes	No
	Nik1p	Cytoplasm	Two H-box domains	Yes	Yes	Yes	No	Yes
	Chk1p	Unknown	Soluble protein, quorum sensing	Yes	Yes	Yes	No	Yes
**Intermediate transfer protein**	Ypd1p	Nucleus and cytoplasm	Intermediary role	Unknown	Yes	Yes	Unknown	Yes
**RR**	Ssk1p	Cytoplasm	Downstream protein of Ypd1p	Yes	Yes	Yes	Yes	Yes
	Skn7p	Nucleus	Related to HSGs	Unknown	Yes	No	No	Yes
	Crr1p/ Srr1p	Cytoplasm and nucleus/ Mitochondria	CUG branch of *Candida*, apoptosis	Unknown	Unknown	Unknown	Unknown	Yes

**HPKs of *C. albicans* (Sln1p, Nik1p/Cos1p, and Chk1p**): Sln1p, a homolog of *Saccharomyces cerevisiae* Sln1, was initially identified in *C. albicans* by Nagahashi et al. [43]. Sln1p consists of 1373 amino acids, including a histidine kinase domain and a C-terminal receptor domain [54], with two transmembrane helices and can rescue the function in *S. cerevisiae SLN1* null strain, indicating that the function of *SLN1* is similar to *ScSLN1*. Nik1p/Cos1p contains 1081 amino acids and is an apparent ortholog of group III histidine kinases [43,54–57]. The NcNik1/Os1p (*N. crassa*), BcBos1p (*Botrytis cinerea*), AbNik1p (*Alternaria Brassicicola*), NcNik1p (*Parastagonospora nodorum*), and ChNik1p (*Cochliobolus heterostrophus*) also belong to this class [57–60]. Nik1p has two H-box domains (H1 and H2) and is considered a cytoplasmic enzyme as it lacks a transmembrane domain [43,61]. The N-terminus of Nik1p contains 9 HAMP (histidine kinases, adenylylcyclases, methyl accepting chemotaxis proteins and phosphatases) domains, where mutations lead to the most severe osmosensitivity and dicarboximide resistance phenotypes [57,62,63]. Although the structure of Nik1p is similar in these different fungi, the roles of orthologous proteins are not identical. For example, CaNik1p has no apparent effect on osmotolerance but is necessary for normal serum-induced hyphal growth [44,55]. The absence of Nik1p resulted in a near-complete loss of virulence in *A. brassicicola* [60]. *P. nodorum NIK1* deletion reduced asexual sporulation *in vitro* [59]. Chk1p is composed of 2471 amino acid residues and contains a specific serine/threonine kinase and GAF domains (cGMP-phosphodiesterase, adenylyl cyclase and a formate hydrogen lyase transcriptional activator) [61]. Chk1p might be a soluble protein as it has neither any trans-membrane hydrophobic domain nor localization signal domain. Currently, the mode of its sensory stimulation is unclear [64].

**Intermediate transfer protein of *C. albicans* (Ypd1p**): Ypd1p serves as an intermediate transfer protein to transfer phosphate groups from HPK to RR and *YPD1* can complement the *S. cerevisiae YPD1* mutation defected functions [65,66]. Ypd1p is localized in both the nucleus and cytoplasm [67] and encodes a protein of 184 amino acids and may regulate the phosphorylation of Ssk1p (cytoplasm) and Skn7p (nucleus) RRs [45,67–69], but the specific mechanism is not fully understood. *YPD1* is the central molecule of the two-component system, and a decrease in *YPD1* activity is expected to compromise fungal fitness, virulence, and viability [70]. *YPD1* inhibition is fatal to *S. cerevisiae* and *C. neoformans* [49,70–72]. However, *C. albicans* can adapt to the continuous activation of Hog1-MAPK triggered by *YPD1* deletion, actively reducing the level of phosphorylated Hog1 [49], indicating that the function of *YPD1* seems to be different among fungal species.

**RRs of *C. albicans* (Ssk1p, Skn7p, and Crr1p/Srr1p**): Ssk1p is a structural homolog of both *S. cerevisiae* Ssk1p and *Schizosaccharomyces pombe* Mcs4p [52]. Ssk1p is located downstream of the Sln1p-Ypd1p pathway and plays a vital role in cell wall biosynthesis, virulence factor regulation, polymorphonuclear neutrophils (PMNs) immune evasion, osmotic stress response, and antioxidative stresses of *C. albicans* [40–42,73,74]. Skn7p is a heat-shock transcription factor of fungi, initially found in *S. cerevisiae*. When cells receive thermal or oxidative stimulation, the signal is transmitted along Sln1p-Ypd1p, eventually phosphorylating Skn7p to regulate gene expression [45,53,68,75–77]. In *C. albicans*, Skn7p plays an essential role in oxidative stress and morphogenesis, but it has less effect upon the maintenance of the cell wall and the osmotic stress response [53,78–80]. *CRR1/SRR1* is a newly discovered RR in the CUG branch of *Candida* [51,81–83]. Bruce et al. [82] reported that it was located in the cytoplasm and nucleus, with little virulence effect, while Mavrianos et al. [51] showed that Srr1p is located within the mitochondria of *C. albicans* and plays an important role in virulence, morphogenesis, apoptosis, osmotic and oxidative stress, etc. [51,83], indicating that the localization and function of Crr1p/Srr1p needs further investigation.

## Functions of *C. albicans* two-component system

### Cell wall integrity

The cell wall is the main organelle of fungi, which determines its viability, cell shape, and interactions with the environment, especially in mediating adhesion and host immune response [84,85]. The differences in cell wall mannan and mannoprotein compositions between yeast and hyphal phases lead to marked differences in the cytokine profiles exhibited by different types of *C. albicans* cells [86]. The RR (Ssk1p) and each type of HPK in the two-component signaling system are critical for cell wall assembly in *C. albicans* [10,40,42–44,55,56,64,87–89]. There are numerous changes in the cell wall structure of *CHK1* mutants, including the truncation of mannan oligosaccharide and β-1,3-glucan (shortened by about 50%) and β-1,6-glucan (increased about four-fold) levels [61,90]. Interestingly, these two glucans are also indirectly regulated by the hyphal-specific gene (RIM101) under different pH conditions [8,91,92]. The killing efficiency of neutrophils to *Candida* was lower when cell wall mannan was added, suggesting that the changes of glucan and mannan in *CHK1* mutants might lead to enhanced PMN response [93,94]. The adhesion and invasion of *SSK1* and *CHK1* mutants to the reconstituted human esophageal tissue (RHE) was lower than that of wild-type strains [38,42], which might be attributed to the changes in cell wall components of *CHK1* mutants and the down-regulation of Als1p [95] of *SSK1* mutants [41,42,96]. Besides, both *SLN1* and *NIK1* mutants altered the transcription levels of some N- and O-mannosyltransferases, suggesting their role in cell wall assembly and maintenance [61].

### Hyphal forms and virulence

Many factors are believed to be related to the virulence of *C. albicans*, including the expression of adhesion molecules (adhesins and extracellular enzymes), immune escape (cell wall mannoprotein and phagocytosis interference), and the morphological transformation of yeast to pseudohyphae/hyphae [9,42,89,97,98]. A complex transcriptional regulatory network controls the morphological transformation of *C. albicans* [8]. Many environmental factors can initiate or inhibit the morphogenetic switch, such as pH, temperature, serum, presence or lack of specific nutrients, etc [80,89,99–101]. Hypha-specific genes (HSGs) have been classified into at least three groups, including transcription factors (*CPH1* and *EFG1*), genes encoding the mitogen-activated protein (MAP) kinase signaling pathway (*MEK1* and *CST20*), and genes expressed only during hyphal growth: hyphal cell wall protein (*HWP1*) and candidalysin (*ECE1*). A basic correlation has been established between hyphal growth defects and virulence [8]. The virulence of *C. albicans* with *SLN1* or *NIK1* deletion is decreased, while the deletion of *CHK1* resulted in loss of virulence, in line with the hyphal defect [44]. The yeast to hyphae transition depends not only on induction conditions but also on the physical state of the medium (solid or liquid) [40,102–106]. The hyphal forms of the *NIK1* mutant cultured in 30°C liquid media could not be distinguished from that of the wild-type strain, while the hypha formation of *NIK1* mutants was defective on a solid agar plate at 37°C [44,55,56,87]. *CHK1* and *SSK1* mutants had a hypha-forming defect on medium 199 (pH = 7.5), Spider medium, and serum-mediated solid medium. However, they developed hyphae and flocculate extensively in liquid media, possibly due to the false expression of proteins on the cell surface [18,40,44,89,107]. *CHK1* mutants can also form hyphae similar to the wild type strain but down-regulate the expression of virulence factors in liquid media when co-cultured with oral epithelial cells, indicating its critical role in oral candidiasis [107]. Interestingly, cell aggregation occurs not only in liquid media, but also on solid media. The *CHK1* mutant formed smooth colonies on solid media probably because the cells aggregated in the colonies and could not grow normally to form the same fuzzy colonies as the wild-type strains [89,90].

**Figure 6.**
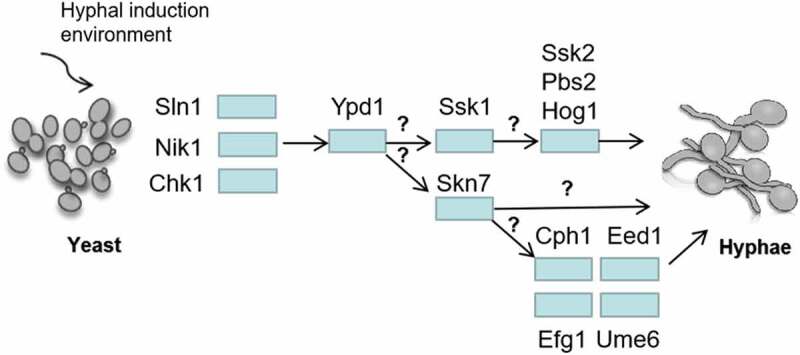
Regulation of *C. albicans* hyphal development by two-component system. Sln1p, Nik1p and Chk1p transfer the regulation signals to RR through Ypd1p. It is still unknown how to distinguish and transmit signals to the downstream RR (Ssk1p and Skn7p). The hyphal forms of *NIK1* mutant cultured in 30°C liquid media is similar with the wild-type strain, while it was defective on a solid agar at 37°C. *CHK1* mutants and *SSK1* mutants have a hyphal formation defect on solid medium, but they can develop hyphae and flocculate extensively in liquid media. The deletion of *YPD1* increased the hyphae formation and flocculation in liquid media. Overexpressing *SKN7* in *EED1, EFG1, CPH1* and *UME6* mutants did not show the similar wrinkled and contained filamentous cells compared to that in wild type strain, suggesting that *EED1, CPH1, UME6* and *EFG1* are essential for *SKN7* function in morphogenesis, but the mechanisms are still unknown

The *SSK1* mutants were defective in hypha development on the nitrogen-rich solid media; however, they formed many hyphae and invaded the solid agar on nitrogen-limited solid media. Therefore, *SSK1* might not be required to develop hyphae but might play a role in hypha regulation [40].The *TPK1*-encoded protein kinase A (PKA) plays a critical role in regulating morphogenesis of *C. albicans* [80,108]. The hyphal formation defect of *TPK1* mutants on solid media was similar to that of *SKN7* mutants [53,80,108], suggesting that *SKN7* may be related to *TPK1* [80], but further evidence is needed to reveal the interaction between them. *SKN7* was also closely correlated with hyphal-specific genes such as *CPH1, EED1, EFG1* and *UME6* [80]. Overexpressing *SKN7* in wild type strains were formed wrinkled colonies and contained filamentous cells. However, the overexpression of *SKN7* in *CPH1, EED1* or *EFG1* mutants did not appear wrinkled colonies and only yeast cells were found, while overexpression of *SKN7* in *UME6* mutants resulted in slightly wrinkled colonies with yeast cells and pseudohyphae [80]. These results suggest that *CPH1, EED1, EFG1* and *UME6* are essential for *SKN7* function in morphogenesis regulation. *SKN7* was closely correlated with hyphal-specific genes such as *CPH1, EED1, EFG1*, and *UME6* [80] (Figure 6). The hypha formation is also related to the accumulation of reactive oxygen species (ROS) [109]. When *C. albicans* are exposed to hypha-inducing solid media, *SKN7* is required to limit the accumulation of ROS [80]. A limited number of studies have evaluated intermediate transfer proteins, but it has been reported that the deletion of Ypd1p increased the hypha formation and flocculation of *C. albicans* in liquid media [67]. However, it seems that decreased virulence was not due to the absence of hyphae *in vivo* [110]. The virulence of *NIK1* mutants is significantly lower than that of the wild-type strain; however, they still form extensive pseudohyphae in tissues [110]. Hypha formation of *SKN7* mutants is defective, but the virulence does not decrease [53,96]. In the downstream MAPK pathway of the two-component system, both *PBS2* and *HOG1* mutants attenuated virulence in a mouse model of disease [111], indicating that *PBS2* and *HOG1* positively regulate the virulence of *C. albicans*. Currently, the regulation of *SSK2* on virulence has not been reported.

The virulence and immune system evasion of *C. albicans* seem to be tissue-specific [42,112]. *CHK1* and *SSK1* mutants are both nontoxic in the disseminated murine model of candidiasis; however, *CHK1* mutants are toxic in the rat model of vaginitis [40,73,87,113]. This could be associated with the difference in pH value between the surface of vaginal mucosa and blood (acid vs neutral) resulting in differential *C. albicans* gene expression at these two sites. Increased production of neutrophil-dependent lactic acid induces cell wall remodeling, masking critical pathogen-associated molecular patterns (PAMPs), such as glucans, blocking immune recognition, and allowing *C. albicans* to colonize and invade the host [114]. PMNs are essential for the host’s resistance to invasive candidiasis, but they are not observed in cell infiltrations of vaginitis in rats [115–117]. Although PMNs are recruited in the vagina, they do not impact the clearance of *C. albicans* [112]. Compared with parental strains, *SSK1* and *CHK1* mutants are more susceptible to growth inhibition and killing efficacy of PMNs [74,116]. The sensitivity of *SSK1* mutants to human neutrophil defensin-1 (HNP-1) was higher than that of wild-type strains [74].

**Figure 7.**
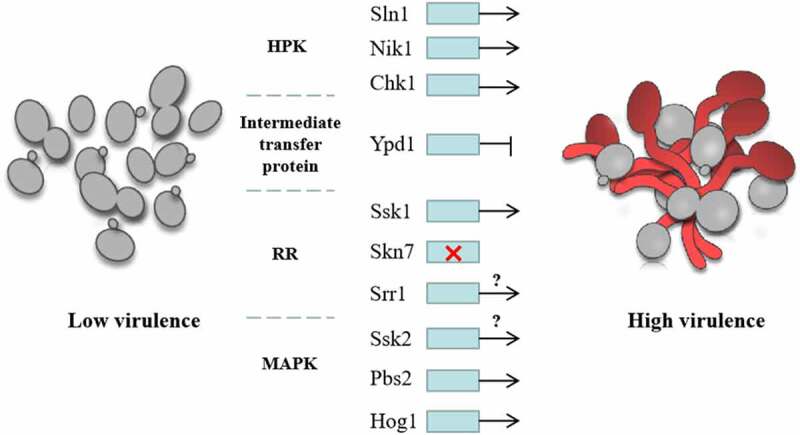
Regulation of *C. albicans* virulence by two-component system. The virulence of *C. albicans* with *SLN1* or *NIK1* deletion is decreased. *CHK1* and *SSK1* mutants are both nontoxic in the disseminated murine model of candidiasis, suggesting that HPKs and *SSK1* positively regulate the virulence of *C. albicans*. The inhibition of the expression of *YPD1* increased the virulence of *C. albicans*, indicating Intermediate transfer protein negatively regulated the virulence. *SKN7* had little effects on the virulence of *C. albicans*, while the regulation of *SRR1* on virulence is unclear. In the downstream MAPK pathway, both *PBS2* and *HOG1* mutants attenuated virulence in a mouse model, indicating that *PBS2* and *HOG1* positively regulate the virulence of *C. albicans*, while the regulation of *SSK2* on virulence is still unknown

It is noteworthy that inhibiting the expression of *YPD1* increases *C. albicans* virulence and its ability to kill macrophages, which might be related to the phenotype of increased hyphae [49]. The *SKN7* mutants were significantly less susceptible to the killing by PMNs than the *SSK1* mutants, and their virulence in the disseminated murine candidiasis model was only mild or not weakened [53]. By knocking out the HPK gene and constructing combinations of single and double mutants, the *CHK1* and *SLN1*, and *CHK1* and *NIK1* double mutants were survivable, while the *SLN1* and *NIK1* double mutants could not be constructed, indicating that the pairing loss of these kinases was a fatal event [44]. Moreover, *C. albicans* lack of Chk1p was nontoxic in the disseminated murine candidiasis model; however, if *CHK1* mutation was accompanied by *SLN1* or *NIK1* deletion, both hyphal development and virulence of the mutant were enhanced [44]. Deletion of both *SSK1* and *HOG1* can negatively regulate the expression of *CHK1* [41,96], suggesting that a complex HPK interaction regulates the development of hyphae and virulence [18] (Figure 7). In addition, the *SSK1* mutant also down-regulated the expression of the following hypha regulation and virulence factors: *HYR1, HWP1, ECE1, MIG1, GCN4, RFG1* (*ROX1), RBF1, RIM101, HAC1, HAP5, TUP1, NRG1, EFG1*, and *CPH1* [73].

### Osmotic stress sensitivity

**Figure 8.**
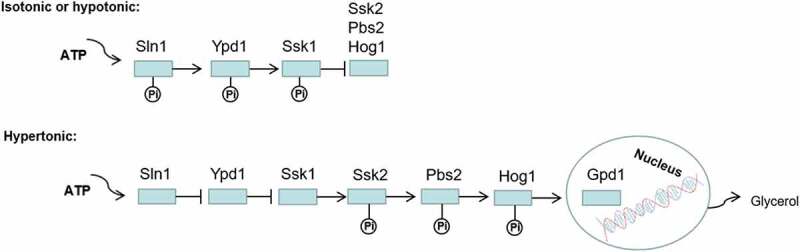
Regulation of osmotic stress response by two-component system. Sln1p acts as an osmotic sensor protein to regulate the Hog1-MAPK signal transduction system in *C. albicans*. When the cells are in an isoosmotic or hypoosmotic environment, phosphorylation of Ssk1p inhibits activation of the Hog1-MAPK cascade, but in hyperosmotic cells, unphosphorylated Ssk1p activates the Ssk2/22 MAPKKK and subsequent phosphorylation of Pbs2p and Hog1p. Finally, the phosphorylated Hog1p is transferred to the nucleus, which activates transcription factors to induce the expression of *GPD1*, increasing the intracellular glycerol content to adapt to hyperosmotic stress

Sln1p serves as an osmotic sensor protein and regulates the Hog1-MAPK signal transduction system in *S. cerevisiae* and *C. albicans*. When the cells are in an iso-osmotic or hypo-osmotic environment, Sln1p first phosphorylates histidine residues in its kinase region and transfers phosphate groups to its aspartic acid residues, phosphorylating the downstream proteins Ypd1p (His) and Ssk1p (Asp). The phosphorylated Ssk1p cannot activate Ssk2p, leading to the shutdown of the Hog1-MAPK cascade. Another situation is that increased extracellular osmolarity deactivates Sln1p without the above-mentioned phosphate transport. A non-phosphorylated form of Ssk1p initiates the downstream signal system and continuously activates Ssk2p/Ssk22p (MAPKKK), Pbs2p (MAPKK), and Hog1p (MAPK). Finally, the phosphorylated Hog1p is transferred to the nucleus, activating transcription factors to induce the *GPD1* expression, which increases the intracellular glycerol content to adapt to hyperosmotic stress (Figure 8). In *SLN1* or *YPD1* mutants of *S. cerevisiae*, constitutive activation of the Hog1-MAPK pathway results in glycerol overproduction and cell death [15,36,69,118–122]. Ssk2p and Ssk22p are redundant proteins in *S. cerevisiae*, while Ssk2 is required for the stress-induced phosphorylation and nuclear accumulation of Hog1 in *C. albicans* [88,123]. There is another *SHO1* upstream branch in the Hog1-MAPK pathway [124]; however, the *SHO1* branch does not significantly affect the activation of the Hog1 pathway in *C. albicans* [125,126].

*C. albicans* can tolerate higher levels of osmotic stresses than many other fungi [127]. Although the absence of Sln1p makes the strain, slightly to moderately, sensitive to osmotic stresses, this mutation is not fatal [18,43]. However, the *NIK1* mutant is not sensitive to osmotic stresses, and its growth is not significantly affected by hypertonic conditions [18,55]. Many phenotypes of *YPD1* mutants depend on the overactivation of *HOG1*, including increased virulence, hyphae, flocculation development, and reduced antioxidant activity [49,67]. It is noteworthy that *S. cerevisiae* Ypd1p can stabilize the Asp phosphorylation of Ssk1p and mediate the retrograde transfer of phosphate from Ssk1p to Sln1p [18,66,69,128], reducing the constitutive lethal activation of the Hog1-MAPK kinase cascade.

### Oxidative stress sensitivity

**Figure 9.**
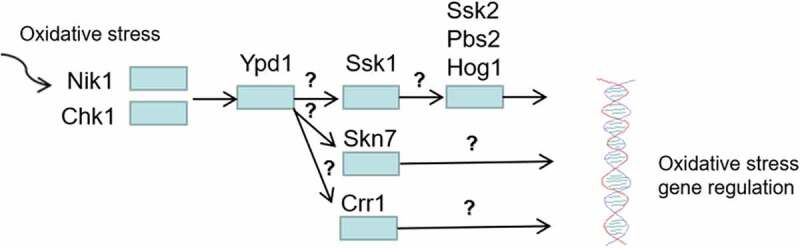
Regulation of oxidative stress response by two-component system. Among the three HPKs Nik1 and Chk1 are required for activation of Ypd1 in response to oxidative stress, then the three RRs (Ssk1, Skn7, Crr1) are activated to regulate oxidative stress by transmitting oxidative stimulation signals to different downstream proteins

Two-component system proteins play an essential role in the oxidative stress response of *C. albicans* (Figure 9). Of the three HPKs, the *CHK1* mutant was the most sensitive to H_2_O_2_, followed by the *NIK1* mutant; the *SLN1* mutant was similar to the wild-type strain [96]. Three RRs (*SSK1, SKN7*, and *CRR1*) are necessary for *C. albicans* to resist oxidative stresses [41,53,80,82]. Cells lacking *SSK1* and *SKN7* are more sensitive to a series of oxidants, including H_2_O_2_ and t-BOOH *in vitro* [41,53,74,80]. The three RRs seem to transmit oxidative stimulation signals to different downstream proteins because the phosphorylation of Hog1p under oxidative stress requires Ssk1p, which is independent of *SKN7* and *CRR1* [41,53,69,80,82]. *SKN7* activation by oxidative stresses requires functional mitochondria in *S. cerevisiae* [41]. The *YPD1* mutant of *C. albicans* was highly resistance to H_2_O_2_ and t-BOOH, but it was very sensitive to sodium arsenite [49,67].

### Quorum sensing

The relationship between cell density and hypha formation of *C. albicans* is similar to the quorum sensing system of some bacterial species [129]. Under the same conditions, *C. albicans* exist in the form of yeast when the cell density is more than 10^6^ cells/ml, while hyphae are formed when the cell density is less than 10^6^ cells/ml [130–132]. Farnesol is an important quorum-sensing molecule of *C. albicans*, which might inhibit biofilm formation by regulating hyphal morphogenesis [132–136]. Chk1p might be the receptor of the farnesol quorum-sensing pathway of *C. albicans* as *SLN1, NIK1*, and *SSK1* mutants respond to farnesol similar to the wild-type strains, while *CHK1* mutants can still form biofilms when farnesol is added [130]; however, the specific transmission mechanism is not clear. Farnesol may be sensed by proteins upstream of Chk1p and activate pathways containing Chk1p [130], as Chk1p is a cytoplasmic protein.

### Antifungal agents

Currently, classic antifungal drugs mainly include echinocandins, polyenes, and azoles [137]. Due to the significant similarity between fungi and human cells in their genome, cell structure, and signal transduction pathways, the side effects and the development of drug resistance limit the application of antifungal agents [4,138]. The polyene antifungal drugs, such as amphotericin B, have serious hepatorenal toxicity, and azole drugs inhibit the p450-dependent enzymes of mammals, causing common adverse drug reactions such as rash, headache, gastrointestinal reactions, and hepatic injury [139]. The biofilm formed by *Candida* on the surface of mucous membranes, dentures, central venous catheters and other medical devices can serve as physical barriers to drug or molecular penetration, making *Candida* inherently resistant to traditional antifungal drugs and host immune responses [140–142]. Therefore, it is imperative to develop new, safe, and effective antifungal agents. The two-component system is important for the virulence and growth of bacteria and fungi, and, importantly, this signal system has not been found in the human genome sequence. Therefore, the new drug developed for the two-component system can effectively fight against fungi without damaging the host cells, making it an ideal antifungal drug target [15,69,87,143–146]. Shivarathri et al. [147] reported that *SSK1* and *HOG1* mutations can restore the susceptibility of clinical strains of the emerging *Candida* species *Candida auris* to amphotericin B and caspofungin. The *SSK1* and *CHK1* mutants of *C. albicans* were highly sensitive to fluconazole and voriconazole, and the sensitivity of *SSK1* mutants to fluconazole was 30 times higher than the *CHK1* mutants, while the sensitivity of *SLN1* and *SKN7* mutants were slightly higher or equal to the wild-type strains [148]. Both rivanol and niclosamide inhibited the two-component signal system of *C. albicans* and caused cell wall defects by inhibiting the hypha formation and growth. They also significantly enhanced antifungal effects when combined with fluconazole [149]. The deletion of all HAMP domains of Nik1p expressed in *S. cerevisiae* could activate Hog1p in the absence of external stimuli, similar to the effect of bactericide treatment [63], suggesting that it might also be a target for antifungal drug development. The deletion of *YPD1* is not fatal to *C. albicans* and even enhances its virulence [49]. These findings question the efficacy of *YPD1* as a broad-spectrum antifungal target.

## Conclusion and prospect

The two-component system plays a vital role in the activities and pathogenicity of *C. albicans*, and significant progress has been made in the past two decades; however, there are still many aspects to be further explored. For example, how are signals distinguished and transmitted to the downstream different RRs after the intermediate transfer protein received stimulation from different HPKs? What other functions of the Crr1p/Srr1p have not been identified? If Crr1p/Srr1p is present in mitochondria, can the HPK bypass Ypd1 in the nucleus and cytoplasm and directly transmit the signal to the RR? The different regulatory functions of the same two-component system under different conditions and the interaction of the system with other transcription factors or signaling proteins are also needs to be investigated. The components of the signal pathway have not been found in the human genome. The development of new drugs against the two-component system can effectively target fungi without damaging the host cells, to greatly reduce the toxic or side effects of antifungal drugs. The two-component system of *C. albicans* therefore provides the ideal therapeutic target of for new antifungal drugs against *C. albicans*.

## Data Availability

Data sharing does not apply to this article as no new data were created or analyzed in this study.
